# The National Organ Matching and Distribution Service

**Published:** 1973-07

**Authors:** S. D. Nelson, H. J. O. White

**Affiliations:** Deputy Director, National Organ Matching and Distribution Service, South Western Regional Transfusion Centre; Renal Transplant Unit, Southmead Hospital


### The National Organ Matching and Distribution Service

S. D. NELSON, M.B., F.R.C.P.I., M.R.C.Path.
Deputy Director, National Organ Matching and Distribution Service, South Western Regional Transfusion Centre
and
H. J. O. WHITE, M.A., M.B., M.Chir., F.R.C.S.
Renal Transplant Unit, Southmead Hospital

In 1963 Calne showed that 6-mercaptopurine (6 M.P.) could prolong the survival of kidney grafts in dogs. Shortly afterwards, it became apparent that the rejection process in human kidney transplants could be moderated by an analogue of 6 M.P., Azathioprine, and transplantation became an accepted procedure in the treatment of renal failure.

As experience grew, the survival of transplants between genetically similar pairs (sibling-sibling or parent-child) was found to be better than when donor and recipient were unrelated (Murray & Barnes 1968, Barnes et al 1971). In the unrelated group there were a proportion of outstandingly successful transplants which might have been due to chance similarity in the antigenic make-up of donors and recipients. There was therefore a need for tests which would identify suitable donor-recipient pairs.

The identification of the Hu-1 (later HL-A) system of leucocyte antigens (Dausset et al 1965, 1966) held promise as a means of predicting tissue similarity. Problems of reproducibility in the leucocyte agglutination technique which Dausset had used, led to the general adoption of a cytotoxic technique for leucocyte typing. In this technique lymphocyte suspensions are tested with a large panel of specially selected sera. Positive reactions are visualised by adding complement (rabbit serum), which unites with antibody molecules fixed to the cells, and permits the cells to be stained with dyes such as trypan blue. The pattern of positive and negative reactions against the serum panel can then be interpreted as the tissue (or HL-A) type. It is presently thought that a person may have up to four HL-A antigens, inheriting two from each parent. The number of possible HL-A phenotypes is very large, and in order to ensure that cadaver kidneys would be given to well matched recipients, dialysis and transplant units in different areas began to co-operate by pooling their tissue typing data, and by allocating their cadaver kidneys to the best matched recipients. Van Rood (1967) proposed the extension of co-operation beyond national boundaries, and through his efforts the Eurotransplant Foundation was established at Leiden in 1968. There quickly followed two further kidney sharing organisations, France Transplant and Scandia Transplant, and in February 1972 a National scheme for the allocation and distribution of cadaver kidneys throughout the United Kingdom came into operation—The National Organ Matching and Distribution Service (N.O.M.S.). This service is based in the Regional Transfusion Centre, Southmead, Bristol, and receives data about prospective kidney recipients and donors. It also arranges the transport of kidneys from one hospital to another as necessary.

The basic data concerning patients awaiting a transplant are forwarded to Southmead from the dialysis centres throughout the country. Details of the patient's name, hospital, ABO group, HL-A type and the presence of cytotoxic antibodies in the patient's serum, together with relevant details of previous transplants, are stored in the South Western Regional Hospital Board's Computer. In addition, an urgency category is recorded—this allows some flexibility in the matching procedure and if transplantation becomes more urgent, the expected waiting time can be reduced at the expense of HL-A matching. The recipient file is updated twice weekly, and a complete list of recipients is printed out. This can be used in the event of a short term breakdown of the computer. Each week every transplant centre receives a list of their own patients showing the data which will be used for matching.

### Procedure when a donor becomes available

Transplantation centres have been established in every hospital board area. When one such centre has a cadaver kidney (or kidneys) which they themselves do not wish to use, they may ask N.O.M.S. to find a suitable recipient. Clinical details of the donor are recorded, including age, cause of death, blood pressure, renal function, ABO group and HL-A type. Only the ABO and HL-A type are used for matching, and this information is fed into the computer which prints out three lists of ten recipients. The first list shows the best matched patients at the donor centre; the second list shows the most suitable recipients in the donor region, that is, those capable of attending a transplant centre within two hours' travelling time. The third list shows the best matched recipients throughout the country. The lists are ordered so that patients who are HL-A identical with the donor come to the top; these are followed by patients for whom the donor presents one mismatch, and after this come patients in the "Urgent" category. Where there is competition between two or more patients for a given place on the list, preference is given to patients whose ABO group is the same as that of the donor (rather than merely ABO compatible). If competition still exists, a patient without cytotoxic (HL-A) antibodies in his serum is preferred—he is less likely to reject a transplant hyperacutely. A final ordering criterion may be necessary, for example, if two patients of the same ABO group, having no cytotoxic antibodies have the same number of mismatches with a donor. In this case the patient who has been waiting longer is given preference.

When kidneys are made available for distribution, they are offered to the doctors responsible for the patients on the recipient list. Quite frequently it is not possible for the first patient on the list to accept the kidney and the duty officers (who are non-medical personnel) offer it for patients in order on the list until someone accepts. They then make the necessary arrangements for transport, and remain on duty while the kidney is en route. After a suitable interval, they check that the kidney was indeed transplanted, and delete the recipient from the active list.

The service provided by N.O.M.S. is directed towards the use of cadaver kidneys, which are removed from the bodies of suitable patients within a short time after death. Patients whose kidneys are most likely to be of use for transplantation are those aged between 10 and 60 years, who die in hospital after a short illness, often after a road accident. There should be little or no hypotension prior to death, nor should there be any evidence of poor renal function. It is also important to ensure that the donor is free of transmissible infections especially hepatitis B and that there are no malignant neoplasms arising outside the brain, since tumour seedlings transplanted with a kidney may flourish in the recipient. If kidneys are to be used for transplantation, they must be removed from the body within 60 minutes and preferably within 45 minutes. The causes of death of donors whose kidneys were made available to N.O.M.S. are shown in Table 1. Injury is the biggest single cause, but cerebral vascular accidents also contribute many donors.

**TABLE 1**

Causes of death in cadaver donors notified to N.O.M.S. during the period 1.2.72—31.12.72.

| | |
|---|---|
| Trauma | 140 |
| Cerebral Vascular Accident | 78 |
| Overdose, self destruction | 16 |
| Cerebral tumour | 16 |
| Vascular occlusion, cardiac arrest | 4 |
| Status epilepticus | 2 |
| Respiratory failure (including asthma) | 4 |
| Other causes | 6 |
| No details given | 18 |
| | 284 |

### The supply of donors

Unfortunately in Britain, the supply of cadaver kidneys does not keep pace with demand although the numbers have increased each year. In 1971, two hundred and five kidney transplants were recorded by the Department of Health, and in the eleven months February-December 1972, the number of transplants given to patients on the N.O.M.S. list was 441. The monthly rate of acquisition of donors shows considerable variation (to date the range is between 22 and 53 per month) and the waiting list which stood at 450 on February 1st, 1972 (not all units had then sent in their patients' details) had risen to 796 by June 1973.

The shortage of donors unfortunately means that occasionally the surgeons are forced to transplant kidneys which they would prefer not to use, because of extreme difficulty in maintaining some patients on dialysis. This use of kidneys which are less than ideal is reflected in their survival after transplantation. Follow up data on the transplants notified to N.O.M.S. in 1972 were collected, and the results for patients receiving their first transplant are summarised in

**TABLE 2**

Survival of 324 first kidney transplants notified to N.O.M.S. during the period 1.2.72—30.11.72.

| | | |
|---|---|---|
| Number of 1st transplants followed up | = 324 | |
| Number functioning at 31.12.72 | = 200 | (61.7%) |
| Number failing before end of 1st week (Includes primary non-function) | = 56 | (17.3%) |
| Number failing during remainder of first three months | = 54 | (16.7%) |
| Number failing after more than three months | = 14 | (4.3%) |

Table 2. There is no way at the moment of distinguishing between kidneys which failed completely to function because they were non-viable (primary non-function) and kidneys which were acutely rejected. If half the kidneys failing before the end of the first week were non-viable (and this seems a reasonable estimate) then one kidney transplant in twelve carried out was doomed to failure from the start. This unsatisfactory state of affairs could be considerably improved if the supply of donors were increased. Most transplant surgeons already do their best to obtain cadaver donors for their own and other units but as Crosby et al (1971) have shown, many more potential kidney donors die in hospital than are notified to the local transplant unit. It would be in the best interests of patients awaiting a transplant, not only locally, but even nationally, if all who care for patients aged between 15 and 50 dying in hospital especially after a period of ventilator respiration were to ask themselves "Could this patient's kidneys be used for transplantation"? If this answer is "perhaps" or "yes" then the local transplant team should be informed before the ventilator is switched off. They would be grateful for this form of help and encouragement from their colleagues.

### REFERENCES

Barnes B. A., Murray J. E. and Atkinson J. C. (1971). "Transplant survival and patient activity data from the human kidney transplant registry". Transplantation Proceedings **3**, 303-307.

Calne R. Y. (1963). "Renal Transplantation"—Arnold, London.

Crosby, D. L., West R. R., and Davies H. (1971). "Availability of Cadaveric kidneys for transplantation" British Medical Journal **4** 401-402.

Dausset J., Ivanyi P., Feingold N. (1966). "Tissue alloantigens present in human leucocytes". Annals of The New York Academy of Sciences **129**, 387-407.

Dausset J., Ivanyi P., and Ivanyi J. (1965). "Tissue alloantigens in humans; identification of a complex system" (Hu-1) pp 51-62 in Histocompatibility Testing 1965). Editors: Balner H., Cleton F. J., and Eernisse J. G., Munksgaard, Copenhagen.

Murray J. E., and Barnes B. A. (1968). "The world-wide status of kidney transplantation" pp 45-60 in Human Transplantation". Editors: F. T. Rapaport and J. Dausset. Grune & Stratton, New York and London.

Van Rood J. J. (1967). "A proposal for international co-operation in Organ Transplantation" pp 451-452 in Histocompatibility Testing 1967. Editors: E. S. Curtoni, P. L. Mattiuz and R. M. Tosi, Munksgaard, Copenhagen.

